# Fortification of milk powder with cashew apple juice using maltodextrin as a carrier material: A novel dairy recipe

**DOI:** 10.1002/fsn3.4390

**Published:** 2024-08-07

**Authors:** Vinoth Kannan Sithu Rameshbabu, Vivek Rangarajan, Sampatrao Dagu Manjare

**Affiliations:** ^1^ Department of Chemical Engineering Birla Institute of Technology and Science‐Pilani, K.K. Birla Goa Campus Zuarinagar Goa India

**Keywords:** cashew apple juice, characterization, fortification, maltodextrin, milk powder, spray drying

## Abstract

Food preservation and fortification pose significant challenges in the fruit and dairy sectors, particularly in developing regions with limited infrastructure and rising production volumes. Cashew apples, rich in antioxidants such as vitamin C and polyphenolic compounds, often go to waste due to their high perishability. In Goa, India, these discarded fruits are used to produce “Feni,” an alcoholic beverage, but broader utilization strategies are still needed. This study introduces a novel approach to extend the shelf life of dairy products like milk powder and enhance their nutritional content by fortifying it with cashew apple juice (CAJ) through spray drying. In order to reduce moisture content during spray drying and to obtain a free‐flowing powder of the final product, maltodextrin was added. Maltodextrin alters the adhesive properties of the fruit juice droplets on surfaces and facilitates the formulation of free‐flowing powder. The key parameters including solubility, bulk density, and glass transition temperature, along with structural analyses such as X‐ray diffraction, field emission scanning electron microscope, and Fourier transform infrared spectroscopy, were evaluated to compare the fortified CAJ milk powder with its commercial counterparts. Experiments determined optimal spray‐drying conditions, achieving a free‐flowing powder at inlet and outlet temperatures of 140 and 60°C, respectively, with a 7% maltodextrin concentration (18 DE). The resulting milk powder displayed a Tg value of 76.7 ± 2.3°C, falling within the acceptable range of 65 to 98°C, demonstrating the feasibility of this fortification method based on the spray‐drying process parameters.

## Graphical abstract

Cashew apples, which are rich in vitamin C and antioxidants, are perishable and often go to waste. In Goa, India, cashew apples that are discarded are used to make an alcoholic drink called “Feni,” but there is a need for more widespread use. This study proposes using spray drying on cashew apple juice (CAJ) to improve the shelf life and nutritional value of dairy products such as milk powder. The solubility, bulk density, thermal characteristics (DSC), and structural investigations (SEM, XRD, and FTIR) were used to compare the fortified CAJ milk powder to commercial products.

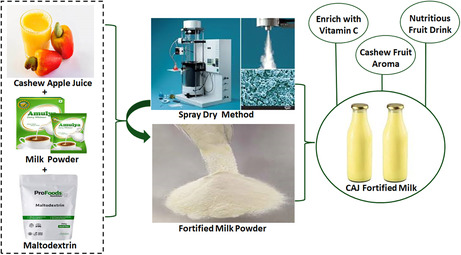

## 1 INTRODUCTION

Dairy milk represents a crucial source of macronutrients, such as calcium, magnesium, phosphorus, vitamin D, vitamin A, riboflavin, vitamin B‐12, zinc, and potassium, for the nutritional requirements of children between the ages of 2 and 8 (Fiorito et al., 2006; Moore et al., 1994). Its consumption significantly contributes to meeting the recommended dietary guidelines for these essential nutrients. On the other hand, alternative beverage choices tend to offer fewer nutrients despite potentially contributing to overall energy intake. Fruit juices are notable sources of substantial amounts of vitamin C and folate. Conversely, consuming sugar‐sweetened beverages, such as soft drinks and fruit drinks, significantly increases daily energy intake and the consumption of added sugars (Murphy et al., 2008). Given these nutritional benefits, the beverage industry increasingly focuses on tailoring products to consumer health interests by exploring nutrient‐rich ingredients. Fruit‐based and dairy beverages are gaining global interest for their protein, calcium, and vitamin contents. Furthermore, the convenience of milk powder, which results from processing liquid milk, satisfies consumer desires with its extended shelf‐life and makes it an ideal candidate for both food product inclusion and international trade (Afifi et al., 2009).

Spray drying is a common technique used in the food industry to produce powders by subjecting items to particular process conditions (Baldelli et al., 2024; Bhandari et al., 1997; Goula & Adamopoulos, 2010; Tontul & Topuz, 2017; Verma et al., 2022). Fruit juice powders provide benefits such as decreased volume and weight, reduced packaging requirements, improved handling, convenient transportation, and significantly extended shelf life. Their consistent physical qualities make them a trustworthy, natural, and quantifiable ingredient used in many food and pharmaceutical items (Fazaeli et al., 2012; Saavedra‐Leos et al., 2021).

Fruit juice powders commonly encounter stickiness, moisture absorption, and lumping challenges due to their high content of low‐weight sugars and acids with low glass transition temperature (Bhandari et al., 1997; Can Karaca et al., 2016). Strategies to mitigate these issues, including using drying agents or additives, optimizing spray dryer configurations, adopting advanced powder processing equipment, and maintaining precise temperature regulators to ensure chamber wall temperatures, remain below the powder's glass transition temperature (Iwuozor et al., 2023; Singh & Verma, 2014). Recent studies have highlighted the use of maltodextrin, liquid glucose, and methylcellulose as drying agents in the food industry, with maltodextrin being the predominant choice for this application (Fazaeli et al., 2012; Singh & Verma, 2014; Sun‐Waterhouse & Waterhouse, 2015; Tonon et al., 2008; Tontul & Topuz, 2017). The inclusion of these ingredients decreases the ability of powder to absorb moisture and elevate the glass transition temperature, representing the threshold point at which a material transitions from a rigid to a pliable state (O'Neill et al., 2019; Saavedra‐Leos et al., 2021). Consequently, these agents are vital in microencapsulation techniques. Such processes offer multiple benefits, including safeguarding sensitive nutritional elements from adverse environmental factors, retaining and protecting aromas and flavors, reducing the volatility of the substance and chemical reactivity, and improving the marketability of the product by enhancing its sensory attributes (Ćujić‐Nikolić et al., 2019). Understanding the glass transition temperature is crucial for predicting the stability and longevity of dry foods. At temperatures below the glass transition temperature, the reduced molecular movements in the solid food structure significantly slow down the degradation processes, such as textural changes, enzymatic breakdown, flavor loss, and nonenzymatic browning (Afifi et al., 2009).

In this context, cashew apples are a seasonal fruit, and making value‐added products available year‐round can be beneficial due to their significant nutritional content, such as carbohydrates (reducing sugars), proteins, and minerals, including phosphorus, calcium, magnesium, iron, zinc, and copper. One method to achieve this is through spray drying, a beneficial microencapsulation technique that helps maintain sensory and biofunctional characteristics of juice constituents in processed products (Baldelli et al., 2024). The powders produced could be utilized in the food business as innovative commercial goods with outstanding sensory attributes, superior quality, little water content, easy transportation, and enhanced shelf life. Furthermore, cashew apple juices are rich in ascorbic acid (Cruz Reina et al., 2022), which can compensate for the lack of vitamin C in milk. Furthermore, the presence of a trace quantity of anacardic acids, which has been tested to possess antiulcerative properties, elevates the application potential of cashew apple juice in the formulation of dairy products (Anoopkumar et al., 2024).

This study aims to develop fortified cashew apple juice (CAJ) milk powder with enriched nutritive value and improved glass transition temperature. The detailed characterization of developed milk powder was carried out using X‐ray diffraction (XRD), field emission scanning electron microscope (FESEM), and Fourier transform infrared (FTIR) spectroscopy to understand crystallinity, surface morphology, and functional properties. Furthermore, the solubility, bulk density, and glass transition temperature of the novel formulated product were estimated.

## 2 MATERIALS AND METHODS

### 2.1 Raw materials

The red‐colored cashew apples (locally identified by the name “vengurla” and specifically cultivated for superior nut qualities) were sourced from Navika Cashew Nursery (15.75° N and 73.86° E) in Mopa, North Goa. The cultivation of these fruits was primarily focused on producing cashew nuts of exceptional grade. After separating the nuts, the fruits (peduncles) were carefully packed and transported to the laboratory in sealed plastic containers. A rigorous cleaning process ensued upon arrival, involving multiple washes with tap water to remove impurities. After being cut into pieces, the juice was extracted with a Bajaj 500 W blender. The extracted juice was stored at 4°C until used. For the powder blend, maltodextrin was procured from Pro Foods Pvt. Ltd., India, and a high‐fat milk powder from Amulya milk powder (Brand: Amul), India, having 20% w/w fat was purchased from a local retailer.

### 2.2 Sample preparation

Prior to spray drying, the cashew apple juice was subjected to a filtration process using a 0.45 μm qualitative filter paper to eliminate larger cashew pulp particles. Following filtration, the juice was fortified with 20% (w/v) milk powder and 7% (w/v) maltodextrin, added to a 500 mL batch of the filtered juice. This mixture was then homogeneously blended under continuous magnetic agitation until all solids were completely dissolved.

Maltodextrin with moderate dextrose equivalent (DE) of 18 DE was used due to its excellent nutritional binding capabilities. Incorporating maltodextrin into the formulation not only increases the concentration of solids but also reduces the moisture content of the final product. Earlier research has shown that a maltodextrin concentration of 7% or higher was required to achieve free‐flowing powder. However, a higher concentration resulted in color loss. Therefore, the current study utilized an equivalent concentration (7%) of maltodextrin to achieve optimal results (Singh & Verma, 2014).

### 2.3 Drying procedure

The spray‐drying process of cashew apple juice was carried out using a laboratory spray drier (Techno Search Instruments, Model: SPD‐P‐111) with a water evaporation rate capacity of 1 L/h, running under co‐current airflow. Co‐current airflow was the optimal direction for food ingredients in this process, minimizing heat damage. The ambient air temperature was kept within the range of 20 to 25°C. The pump's rotating speed was modified to regulate the flow rate of the mixture being supplied to the primary chamber. The drying airflow rate was consistently set at 73 m^3^/h, with a steady air pressure of 0.06 MPa from the compressor. The process parameters followed by Singh and Verma's (2014) recommendations, with optimized conditions including an inlet temperature of 140°C, a feed rate of 1.8 mL/min, and an outlet temperature of 60°C. Atomization of the feed was achieved using compressed air, and resulting droplets were dried within the chamber by hot‐air aspiration. The cyclone air separator aided in the recovery of powder, with dry samples recovered from the bottom of the cyclone. The spray‐dried enriched milk powder samples were discovered to be hygroscopic. Desiccators were utilized to store spray‐dried samples at room temperature ranging from 20 to 25°C.

#### 2.3.1 Yield of spray‐dried powders

The yield (%) of the spray‐dried powder was calculated from the given Equation (1), as the ratio between the total mass of the product recovery and the mass of the extract fed in the system (Tolun et al., 2016).

(1) $$\text{Yield}\;\left(\%\right)=\frac{\text{Mass of the product}}{\text{Mass of feed solution}}\times 100$$

### 2.4 Powder characterization

#### 2.4.1 Bulk density and solubility

The powder properties of spray‐dried powders were assessed using measurements of solubility and bulk density. The method described by Verma et al. (2022) was used in measuring solubility with some adjustments. This procedure involves adding 1 gram of powder sample to 100 mL of distilled water in a blender container. The mixture was stirred at 15,000 rpm for 5 min and then spun in a centrifuge at 2600 rpm for another 5 min. A 25 mL portion of the resulting supernatant was then moved to a preweighed Petri dish and dried in an oven at 105°C for 5 h. The solubility percentage was determined by comparing the weight of the dried sample to the weight of the empty preweighed Petri dish.

The determination of bulk density (g/mL) involves placing a precisely measured 2 g of cashew juice‐fortified milk powder into a graduated 10 mL cylinder. The cylinder is then vibrated using a vortex vibrator for 1 min to obtain an accurate reading. The ratio of the mass of the powder to the volume of the cylinder occupied by the powder is used to determine the bulk density value (Fazaeli et al., 2012).

#### 2.4.2 FESEM analysis

The microstructures of both cashew apple juice‐fortified milk powder and commercial powder (used as reference) were examined using a field emission scanning electron microscope (FESEM, Quanta FEG 250). The powder samples were affixed to stubs with double‐sided adhesive tape and then coated with gold using a sputter‐coating apparatus (LEICA EM ACE 2000) to create a reflecting surface for the electron beam. Afterward, the samples covered with gold were examined under the microscope (Watharkar et al., 2021). The average particle size of commercial and fortified milk powder samples was calculated using ImageJ software (USA).

#### 2.4.3 Glass transition temperature (Tg)

The glass transition temperature (Tg) of spray‐dried powder was measured using a differential scanning calorimeter or DSC (Discovery SDT 650 analyzer, TA Instrument, Waters Pvt Ltd). Samples (10 mg) were subjected to scanning from 0 to 200°C at a heating rate of 10°C per minute under a nitrogen atmosphere (O'Neill et al., 2019). The Tg value was calculated using the TA instrument analysis software TRIOS (V5.2.2.47561).

#### 2.4.4 X‐ray diffraction (XRD)

X‐ray diffraction analysis of fortified cashew apple juice milk powder and commercial milk powder was conducted using an X‐ray diffractometer (Bruker D8 Advance, Germany). The operational conditions were adhered to as outlined by Watharkar et al. (2021). The input energy used was 30 mA current and 35 kV voltage. The sample was scanned within a diffraction angle (2θ) range of 5° to 50° with an incremental step of 0.02°, at a scanning rate of 1 s per step.

#### 2.4.5 Fourier transform infrared (FTIR) spectroscopy

The structural properties of cashew apple juice‐fortified powder and commercial milk powder were determined by attenuated total reflectance–Fourier transform infrared spectrophotometer (ATR‐FTIR, Perkin Elmer, model: Spectrum two, USA) in the wavelength range of 4000–600 cm^−1^ at a resolution of 4 cm^−1^ (Kalušević, Lević, Čalija, Milić, et al., 2017).

### 2.5 Statistical analysis

All studies were performed in triplicate and the average of the three experimental data along with a standard deviation of ±5% limit is reported.

## 3 RESULTS AND DISCUSSION

The cashew apple tree is part of the *Anacardiaceae* family and produces a soft, fibrous fruit that is full of nutritious juice. This juice contains an enormous amount of fermentable sugars, minerals, and vitamins, making it an excellent candidate for the production of cashew apple‐related products. Despite the fruit's high vitamin C content and exceptional antioxidant properties, it is often discarded because of its perishable nature. This has resulted in a waste problem for cashew growers, who struggle to dispose of the by‐products of cashew processing, including the cashew apple, pulp, shell, nut oil, and shell itself (Kannan et al., 2021). Goa is the only Indian state where cashew apples are extensively used to produce an alcoholic beverage called “Feni,” which has received geographical indication (GI) status for its production from cashew apple juice through fermentation (S R et al., 2024). While most cashew apple products are obtained through fermentation, some value‐added end products, such as bio‐surfactants, organic fertilizer, alcohols, esters, and food products (jams, jellies, pickles, energy drinks, and cookies), directly utilize the fruit juice or pulp for manufacturing (S R et al., 2023).

With the increasing interest in sustainable and zero‐waste solutions, more research and investment into processing and utilizing cashew apples can provide new opportunities for the industry. Cashew apple juice‐fortified milk powder, for example, is an innovative product that can be developed from cashew apples to cater to the growing demand for plant‐based milk alternatives. By leveraging the nutritional benefits and natural sweetness of cashew apples, the production of cashew milk powder can offer a sustainable and nutritious alternative to traditional dairy milk. This presents an exciting avenue for further exploration and development in the cashew industry. In this study, we utilized milk powder in combination with freshly extracted cashew apple juice to produce fortified milk powder by spray‐drying method. Additionally, the key physical characteristics of the cashew juice‐fortified milk powder, including bulk density, solubility, surface morphology, glass transition temperature, FTIR, and XRD, were assessed and compared with those of commercial milk powder.

### 3.1 Bulk density and solubility

The powder bulk density is used to assess the economic and practical feasibility of the dry powder by providing a measure of its density. This crucial assessment can provide insights for determining appropriate packing materials, transportation methods, marketing tactics, and the influence of external variables on powdered products (Watharkar et al., 2021). One major factor affecting spray‐dried powder's bulk density and moisture content is maltodextrin concentration. A recent study found that higher levels of maltodextrin can decrease powder density and increase moisture content during spray drying due to the large size of maltodextrin molecules that make it difficult for water to permeate through them. However, augmenting the concentration of maltodextrin can also have a negative impact on health and diminish the product's nutritional value. In view of above discussion, in this study, low concentration of maltodextrin 7% (w/v) was used to avoid the said adverse effects (Sun‐Waterhouse & Waterhouse, 2015).

According to Goula and Adamopoulos (2008), maltodextrins can alter the adhesive properties of fruit juice droplets on surfaces and facilitate the formation of fruit juice powders that flow freely while also minimizing the accumulation of particles on the chamber walls during spray drying. The molecular weight of maltodextrins determines their functional qualities (Sun‐Waterhouse & Waterhouse, 2015), with lower DEs resulting in a higher glass transition temperature and less caking but also a slower evaporation rate due to more viscous feed mixtures. Higher DEs, on the other hand, lead to higher bulk density but also increased stickiness of the mixture due to a lower Tg value (Goula & Adamopoulos, 2010). Table 1 shows the range of bulk density values for the various fruit juice powders with different carrier materials as the drying agent.

**TABLE 1:** Bulk density value for the various spray‐dried juice powders.

Carrier type	Juice sources	Bulk density (g/mL)	Reference
Maltodextrin (4–7 DE)	Orange	0.9 ± 0.1	Baldelli et al. (2024)
	Mango	0.3 ± 0.2	
	Strawberry	0.3 ± 0.1	
Maltodextrin (16.5–19.5 DE)	Orange	1.0 ± 0.1	Baldelli et al. (2024)
	Mango	0.3 ± 0.1	
	Strawberry	0.3 ± 0.1	
Maltodextrin	Sugarcane	0.33–0.79	Iwuozor et al. (2023)
Liquid glucose	Orange	0.5–0.8	Chegini and Ghobadian (2007)
Maltodextrin	Orange	0.21–0.5	Chegini and Ghobadian (2007)
Maltodextrin (6, 12, 21 DE)	Orange	0.14–0.4	Goula and Adamopoulos (2010)
Maltodextrin (6, 12, 20 DE)	Black Mulberry	0.35–0.55	Fazaeli et al. (2012)
7% Maltodextrin	Mosambi	0.39	Singh and Verma (2014)
7% Maltodextrin (18 DE)	Cashew Apple	0.36 ± 0.03	Current work

A recent study conducted by Baldelli et al. (2024) revealed that when maltodextrin (4–7 DE) was used as a carrier agent, bulk densities of the final powder were in range of 0.3 to 0.9 g/mL for different fruit juices such as orange, mango, and strawberry. In a similar work, the bulk density of spray‐dried powder obtained from sugarcane juice varied between 0.33 and 0.79 g/mL when maltodextrin was employed as a drying agent (Iwuozor et al., 2023). In another work, maltodextrin and liquid glucose were used as drying agents to produce orange juice powder from juice concentrates. The resulting powders showed a bulk density ranging from 0.5 to 0.8 g/mL when liquid glucose was employed as a drying agent. On the other hand, the powders obtained using maltodextrin as a drying agent exhibited a lower density, ranging from 0.21 to 0.5 g/mL (Chegini & Ghobadian, 2007). In another study, the authors reported that the bulk density of spray‐dried concentrated orange juice varied between 0.14 and 0.40 g/mL for various ratios of orange juice to maltodextrin (6, 12, and 21 DE) (Goula & Adamopoulos, 2010). The bulk density for mosambi juice powder was found to be 0.39 g/mL with 7% maltodextrin as the carrier agent (Singh & Verma, 2014). The black mulberry juice powders have bulk densities ranging from 0.35 to 0.55 g/mL when various carrier agents, including maltodextrin (6, 9, and 20 DE) and gum Arabic, were used (Fazaeli et al., 2012). In the present work, the bulk density of cashew apple juice‐fortified milk powder was determined to be 0.36 ± 0.03 g/mL using maltodextrin concentration of 7% as compared to commercial milk powder which was found to be 0.30 ± 0.01 g/mL. These findings were corroborated by the other reported values for different substrates and drying agents.

As maltodextrin's DE increases, the solubility of the resulting powder decreases. The solubility of the powder is affected by the moisture level, which is determined by the maltodextrin DE. This phenomenon can be explained by the correlation between low moisture content and rapid rehydration. Goula and Adamopoulos (2008)) reported that a reduced moisture content results in a less sticky powder, which in turn increases the surface area in contact with the rehydration water. This study demonstrates that the solubility of the maltodextrin (18 DE) used in this experiment reaches a maximum of approximately 71 ± 1.8%. Previous studies used maltodextrin with a DE of 12 to make kiwifruit juice–milk powders that have low water activity, low hygroscopicity, and high solubility. These powders contain a considerable quantity of hydrophilic groups and have a relatively slow drying rate (Sun‐Waterhouse & Waterhouse, 2015). In a recent study, it was shown that the solubility of sugarcane juice powder with maltodextrin as a carrier agent ranged from 81.05% to 98.06%, depending on the inlet and outlet temperatures used in the spray‐drying process (Iwuozor et al., 2023). In another work, the solubility range of the black mulberry juice powder is between 80% and 88%, and it can be achieved by using different carrier agents such as 8% maltodextrin (with a DE of 20, 9, or 6) and gum Arabic with an inlet temperature of 130°C (Fazaeli et al., 2012). The solubility level achieved in this experiment was 71 ± 1.8% at an inlet temperature of 140°C for CAJ‐fortified powder and 83 ± 1.3% was observed for the commercial milk powder. The inlet temperatures are within the same range as those recorded in the current investigation. Conversely, around 87% of the highest powder solubility was achieved by blending 6% gum Arabic and 2% maltodextrin 6 DE (Fazaeli et al., 2012). Moreira et al. (2009) observed similar findings using spray‐dried acerola pomace extract. Another study involved incorporating nano‐curcumin into milk powder using different spray‐drying temperatures and encapsulating materials such as sodium caseinate and gum Arabic. The solubility of the resulting product ranged from 46.92% to 52.01% (Verma et al., 2022). Table 2 shows the range of solubility values for the various fruit juice powders with different carrier materials as the drying agent.

**TABLE 2:** Solubility values for the various spray‐dried juice powders for different juice sources.

Carrier type	Sources	Solubility (%)	Reference
Maltodextrin	Sugarcane	81.05–98.06	Iwuozor et al. (2023)
8% Maltodextrin (6, 9, and 20 DE) and gum Arabic	Black mulberry	80–88	Fazaeli et al. (2012)
6% gum Arabic and 2% maltodextrin 6 DE	Black mulberry	87	Fazaeli et al. (2012)
6% gum Arabic and 2% maltodextrin 6 DE	Acerola pomace extract	87	Moreira et al. (2009)
Sodium caseinate and gum Arabic	Nano‐curcumin MP	46–52	Verma et al. (2022)
7% Maltodextrin 18 DE	CAJ‐MP	71 ± 1.8	Current work

### 3.2 Process yield

The yield of the spray‐drying process is defined as the ratio of the mass of powder recovered by the mass of total solids in the feed, which is a significant indicator from an industrial perspective (Tolun et al., 2016). The primary factor contributing to the low product yield during the spray‐drying process is mostly attributed to the adhesive properties of the food constituents (Can Karaca et al., 2016). The variation in yield also depends on the type and quantity of fruit in the slurry, as well as the specific experimental settings, employed in each study, with a minimum yield of 50% considered to be economically viable (Bhandari et al., 1997; Zareifard et al., 2012). In our study, the process yield of cashew apple juice‐fortified powder is estimated as 78% with 7% maltodextrin (18 DE) as carrier agent and an inlet temperature of 140°C. The study conducted by Can Karaca et al. (2016) found that the yield of sour cherry powder varied between 23% and 92% with varying dry matter (25% and 50%) when maltodextrin DE 12 employed as a carrier material with the same inlet temperature of 140°C. However, Goula and Adamopoulos (2010) reported that a yield of 95% was obtained for the orange juice powder utilizing maltodextrin (6 DE) as a drying agent. In a similar work, Horuz et al. (2012) and Vardin and Yasar (2012) achieved yields of 86% and 76% using maltodextrin (6 and 7 DE), respectively. Fazaeli et al. (2012) reported that a yield of 82% was achieved for black mulberry juice powder produced with the combination of 6% maltodextrin 6 DE and 2% gum Arabic.

### 3.3 Surface morphology

**FIGURE 1:**
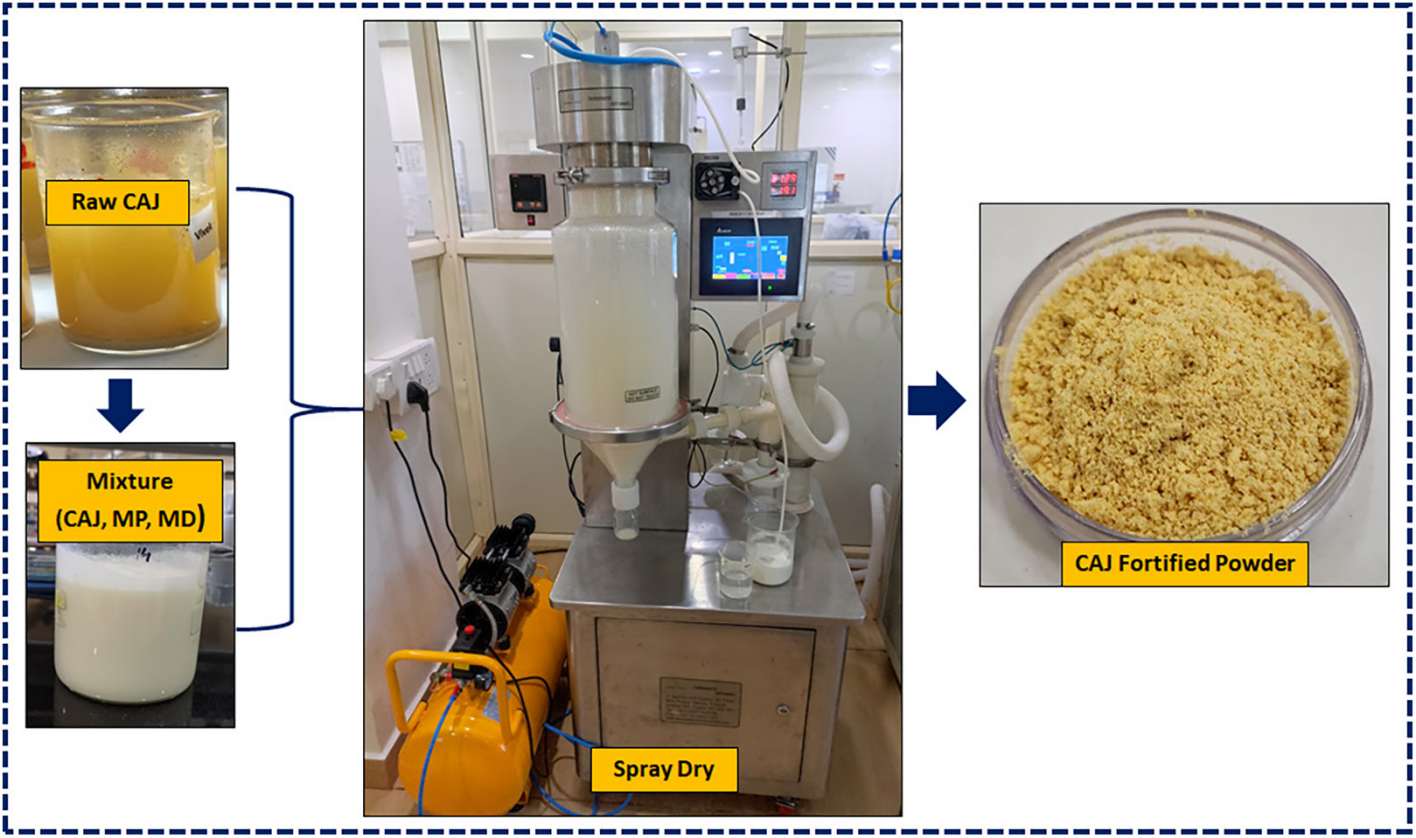
Free‐flow powder of CAJ‐fortified milk powder at 7% maltodextrin, 140°C.

**FIGURE 2:**
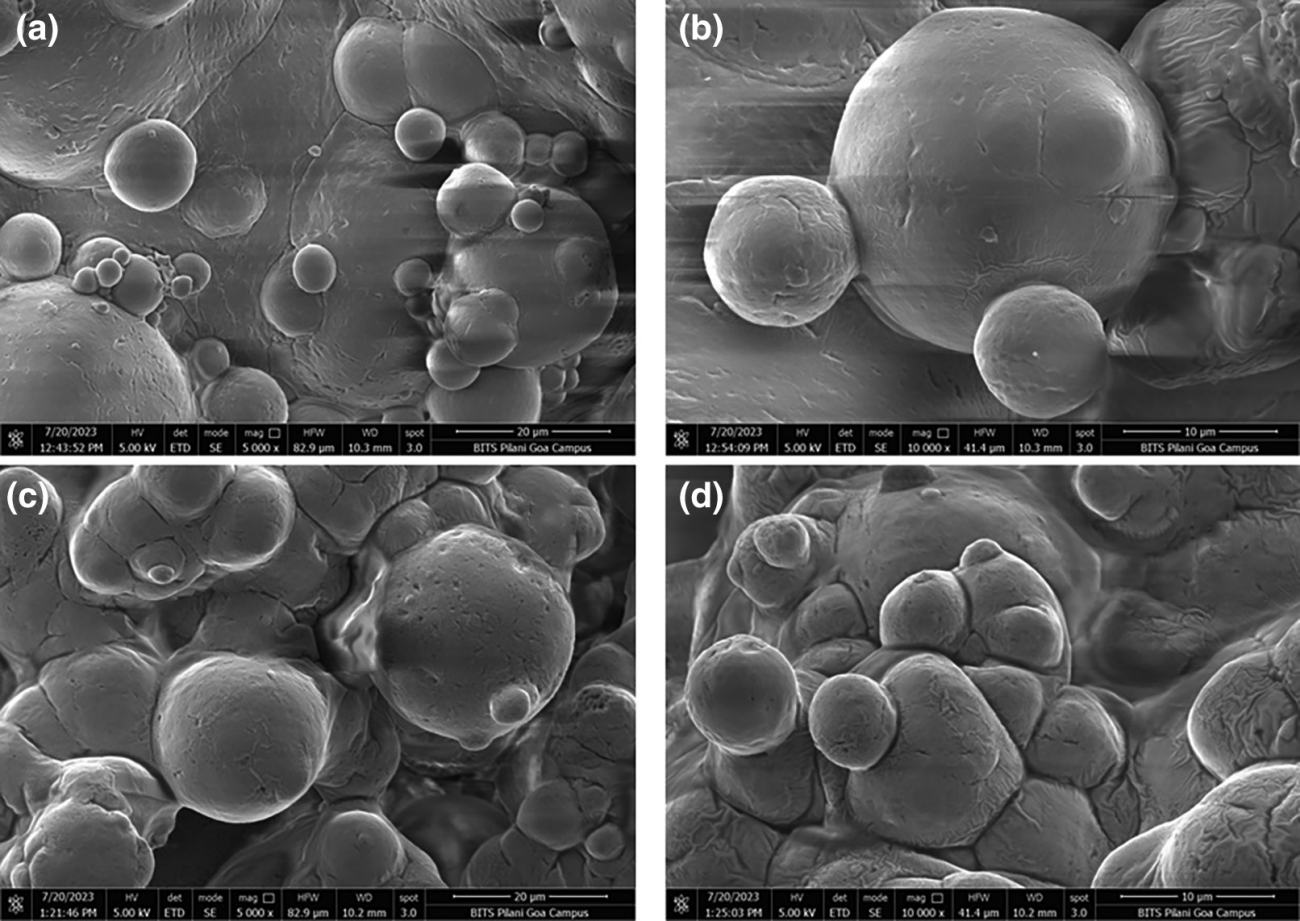
FESEM micrographs of commercial milk powder (C‐MP) and cashew apple juice‐fortified milk powder (CAJ‐MP) in different magnifications with 140°C inlet temperature. (a) C‐MP, 5000x; (b) C‐MP,10,000x; (c) CAJ‐MP, 5000x; and (d) CAJ‐MP, 10,000x.

Figure 1 illustrates the process of CAJ‐fortified milk powder produced in this study (MP—milk powder and MD—maltodextrin). The morphology of spray‐dried microparticles of both commercial milk powder (used as reference) and cashew apple juice‐fortified powder was studied using the FESEM technique (Figure 2a–d). The fortified milk powders were produced with an inlet temperature of 140°C and 7% of maltodextrin (18 DE) in the current work. The FE‐SEM investigation revealed that the CAJ‐fortified powder made with 7% maltodextrin 18 DE had bigger, amorphous particles stacked together and had considerable interparticle interaction (Figure 2c,d). The average particle size of the commercial milk powder was 8.06 ± 3.2 μm, whereas the fortified CAJ milk powder had an average particle size of 5.71 ± 1.78 μm and these findings align with the previously published data. These results corroborated earlier research when powders were produced with maltodextrin 20 DE. The mean particle size of powders varied from 4.4 ± 0.08 to 12.35 ± 0.23 μm for the use of various DEs of maltodextrin (6, 9, and 20 DE) as a carrier agent employed in the production of mulberry juice powder (Fazaeli et al., 2012). In a similar finding with chokeberry fruit extract and chokeberry waste extract, the particle size varied from 4.72 to 11 μm when employing maltodextrin and skimmed milk as carrier agents (Ćujić‐Nikolić et al., 2019). In general, it has been observed that the choice of the carrier material showed a profound influence on the final morphology of the particles. The use of 6% maltodextrin 6 DE with the combination of 2% gum Arabic and 8% maltodextrin 20 DE as the carrier agent increased the average particle size of the mulberry juice formulation from 4.4 ± 0.08 to 12.35 ± 0.23 μm (Fazaeli et al., 2012).

In another work, açai powder was produced via spray drying at three distinct inlet temperatures, utilizing 20% maltodextrin as the drying agent. The resulting particles exhibited a spherical shape and varied in size, consistent with typical materials generated through spray‐drying processes. The research further noted that particles displayed a wrinkled surface at lower inlet temperatures, while those produced at higher temperatures exhibited a smoother surface (Tonon et al., 2008). This observation was corroborated by Figure 2a,b, depicting commercial milk powder, which aligned with the reported findings. The rationale behind this phenomenon lies in the rapid evaporation of moisture at higher drying temperatures, leading to the formation of a dry and rigid outer layer on the particles. Hollow particles cannot collapse when vapor condenses inside the empty space as the temperature decreases in the dryer. Conversely, at lower drying temperatures, the outer layer of the particles retains moisture for a longer duration, allowing hollow particles to deflate and shrink as they cool. The differences in powder morphology at various temperatures can be linked to the varying physical characteristics of the crust, which can range from flexible and compressed at lower‐to‐moderate temperatures to porous at higher degrees (Tonon et al., 2008).

Our findings demonstrate that microparticles encapsulated with cashew apple juice milk powder using maltodextrin (18 DE) as an encapsulating agent exhibit favorable properties, as illustrated in Figure 2c,d. The powder particles produced in our study at 140°C inlet temperature appeared larger and more spherical with fewer surface imperfections. Similar results were observed when employing maltodextrin (18 DE) and sodium caseinate as encapsulation materials at various inlet temperatures (150 and 170°C) (Himmetagaoglu & Erbay, 2019). The incidence of surface imperfections, such as damaged or cracked particles, in the fortified milk powder was minimal in our investigation. Particles that are bigger and have a consistent spherical shape usually show improved powder flow because they have less contact area, specific surface area, interparticle adhesion and friction force (Meena et al., 2018). The presence of dents has a negative impact on the flow characteristics of powder particles. Numerous researchers have reported the observation of spherical particle shapes with smoother surfaces when maltodextrin was utilized as a carrier agent (Kalušević, Lević, Čalija, Pantić, et al., 2017; Kalušević, Lević, Čalija, Milić, et al., 2017; Tolun et al., 2016).

### 3.4 Glass transition temperature (Tg)

**FIGURE 3:**
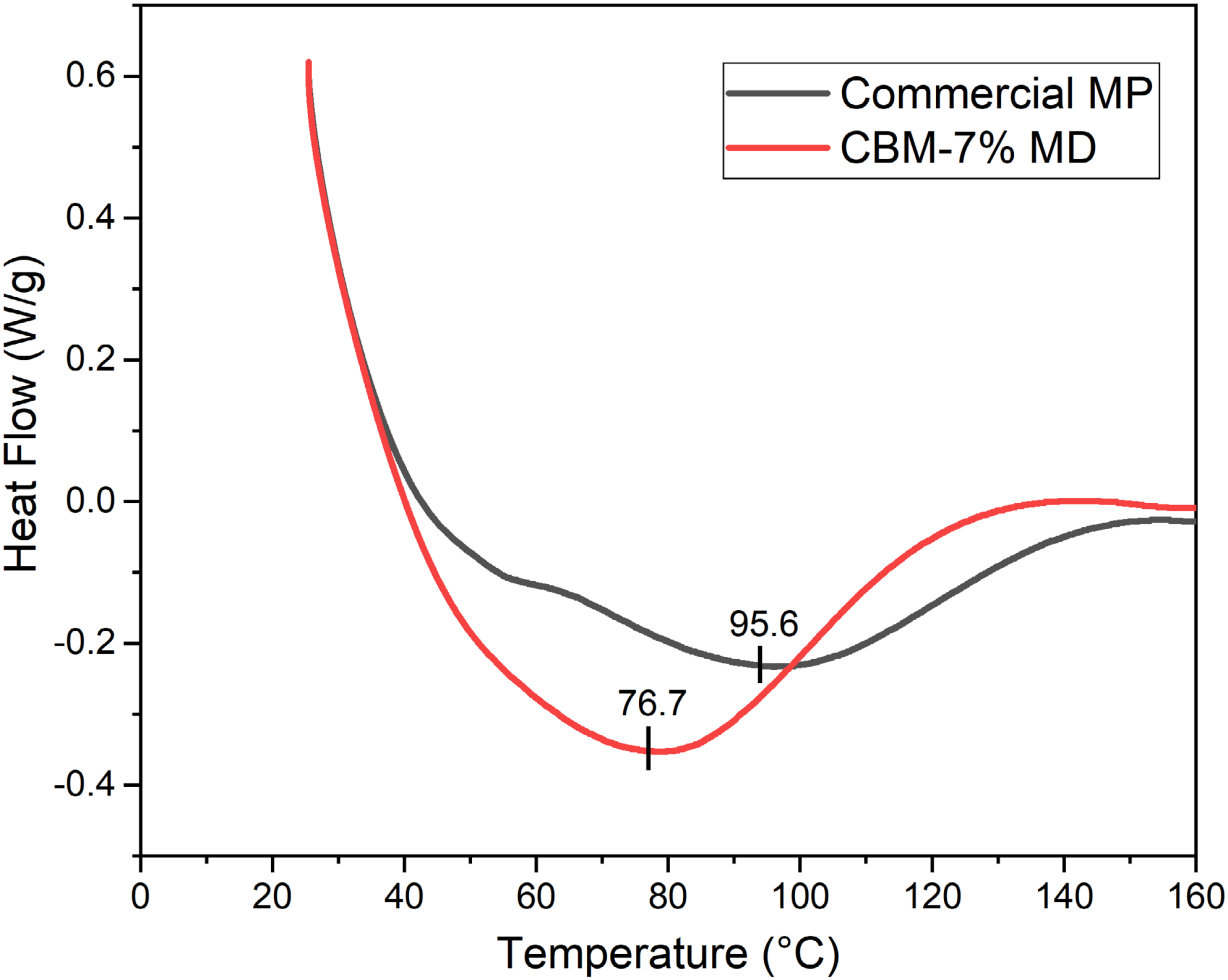
DSC profile for spray‐dried CAJ‐fortified milk powder with the carrier agent of 7% maltodextrin (CBM—7% MD—red) and commercial milk powder (black).

The present research investigated the impact of incorporating maltodextrin (DE 18) on the glass transition point (Tg) of spray‐dried cashew juice‐fortified milk powder. Tg denotes the critical temperature at which the amorphous phase undergoes a significant transition, transitioning from a rigid, crystalline state to a soft, viscous, liquid state. Differential scanning calorimetry (DSC) was utilized to measure Tg, identifying alterations in heat flow within the noncrystalline portion during the transition between the glassy and rubbery phases. Thermograms depicting the relationship between heat flow and temperature were obtained using DSC (Figure 3). The powder produced with 18 DE maltodextrin at a concentration of 7% exhibited a glass transition temperature (Tg) of 76.7 ± 2.3°C, and Tg value of 95.6 ± 2.1°C was obtained for the commercial milk powder in the current work. Table 3 shows the Tg values obtained for various spray‐dried juices by different carrier materials as the drying agent. Since the use of various carrier materials resulted in products of varying consistencies, the choice and the concentration of carrier material play a significant role. The amount of carrier material (maltodextrin) to be added is dictated by its dextrose equivalency, which is inversely related to its average molecular weight. An elevation in maltodextrin's dextrose‐equivalent results in a reduction in the glass transition temperature (Tg) of the powder because of the shorter chains in maltodextrins with lower molecular weight (Kasapis, 2005).

**TABLE 3:** Glass transition temperature (Tg) of various spray‐dried juice powders with different drying agents.

Carrier composition	Juice sources	Inlet temperature (°C)	Tg (°C)	Reference
50% Maltodextrin 6 DE	Orange	160	66.4	Shrestha et al. (2007)
60% Maltodextrin 6 DE	Orange	160	86.4	Shrestha et al. (2007)
8% Maltodextrin 6 DE	Black mulberry	130	73.8	Fazaeli et al. (2012)
6% Maltodextrin and 2% Gum Arabic	Black mulberry	130	76.4	Fazaeli et al. (2012)
Maltodextrin 12 DE	Sour cherry	150	60	Can Karaca et al. (2016)
5% Maltodextrin	Broccoli	150	51	Saavedra‐Leos et al. (2021)
7% Maltodextrin 18 DE	Cashew apple	140	76.7 ± 2.3°C	Current study

Shrestha et al. (2007) performed a study on spray drying of orange juice concentrate with maltodextrin. The glass transition temperature (Tg) of 66.4°C was observed while employing a 50:50 ratio of anhydrous orange juice to maltodextrin with a 6 DE value. However, increasing the maltodextrin content from 50 to 60 parts led to a higher Tg value of 86.4°C. In another work, black mulberry juice was subjected to spray drying using different combinations of carrier materials as the drying agent, with an inlet air temperature of 130°C. The study revealed that the glass transition temperature increased from 73.8 to 76.4°C by combining 6% maltodextrin and 2% gum Arabic as the drying agent (Fazaeli et al., 2012). Conversely, the experiment involved examining the usage of maltodextrin 12 DE as a drying agent for the juice of sour cherry and employing a carrier material of 5% maltodextrin for spray drying the broccoli juice. The Tg values of 60 and 51°C, respectively, were reported in these studies (Can Karaca et al., 2016; Saavedra‐Leos et al., 2021). O'Neill et al. (2019) conducted a study to examine how the addition of maltodextrin (DE 6) and glucose syrup (DE 39) affects the glass transition point and surface‐free fat of spray‐dried emulsions. The study found that adding maltodextrin and glucose syrup as carrier agents at a concentration of 15% (w/w) increased the glass transition temperature (Tg) to 74 ± 6.3 and 71 ± 2.8°C, respectively. The Tg value of 76.7 ± 2.3°C reported in this study pertains specifically to the fortified cashew juice–milk powder produced using 18 DE maltodextrin. These findings are consistent with the Tg observations of Krishnan et al. (2005) and Fazaeli et al. (2012).

### 3.5 X‐ray diffraction (XRD)

**FIGURE 4:**
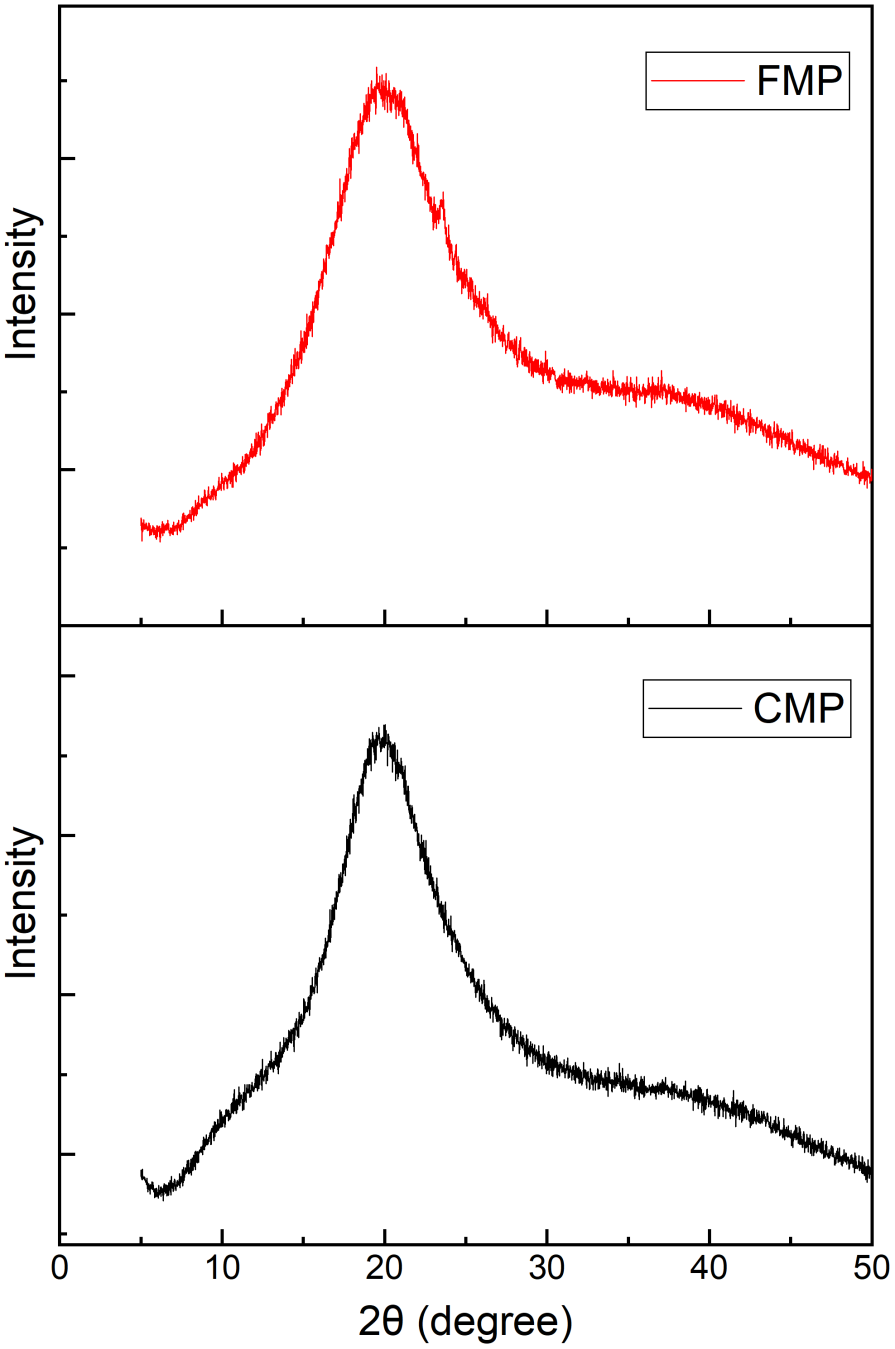
X‐ray diffraction pattern of cashew apple juice‐fortified milk powder (FMP—red) and commercial milk powder (CMP—black).

X‐ray diffraction was carried out to characterize the structure of the spray‐dried cashew apple juice‐fortified powder and the commercial milk powder. In general, disordered molecules in amorphous material led to diffused and prominent peaks, resulting in dispersed bands. On the other hand, crystalline materials produce sharp and well‐defined peaks due to their highly ordered structures (Watharkar et al., 2021). From Figure 4, it is revealed that milk powders, cashew apple juice‐fortified milk powder (FMP‐Red), and commercial milk powder (CMP‐Black) exhibited an amorphous form, as indicated by the presence of diffused peaks with significant noise. During the drying process, the protein present in the milk powder hinders the sugars' ability to establish hydrogen bonds between proteins and reducing sugars, which affects crystallization. The above results (Figure 4) are in alignment with findings reported in the literature which used maltodextrin as a drying agent for spray‐dried mango juice powder and freeze‐dried mango powder (Cano‐Chauca et al., 2005; Harnkarnsujarit & Charoenrein, 2011). In another study, the author found that inadequate drying time can impede the transformation of low‐molecular‐weight amorphous sugars, such as glucose, fructose, sucrose, and organic acids found in fruit juice, into a crystalline state. The amorphous characteristics of cashew apple juice‐fortified milk powder in this work may be attributed to the shorter drying period, consistent with findings from previous research on foam‐dried banana powder (Watharkar et al., 2021).

### 3.6 Fourier transform infrared (FTIR) spectroscopy

**FIGURE 5:**
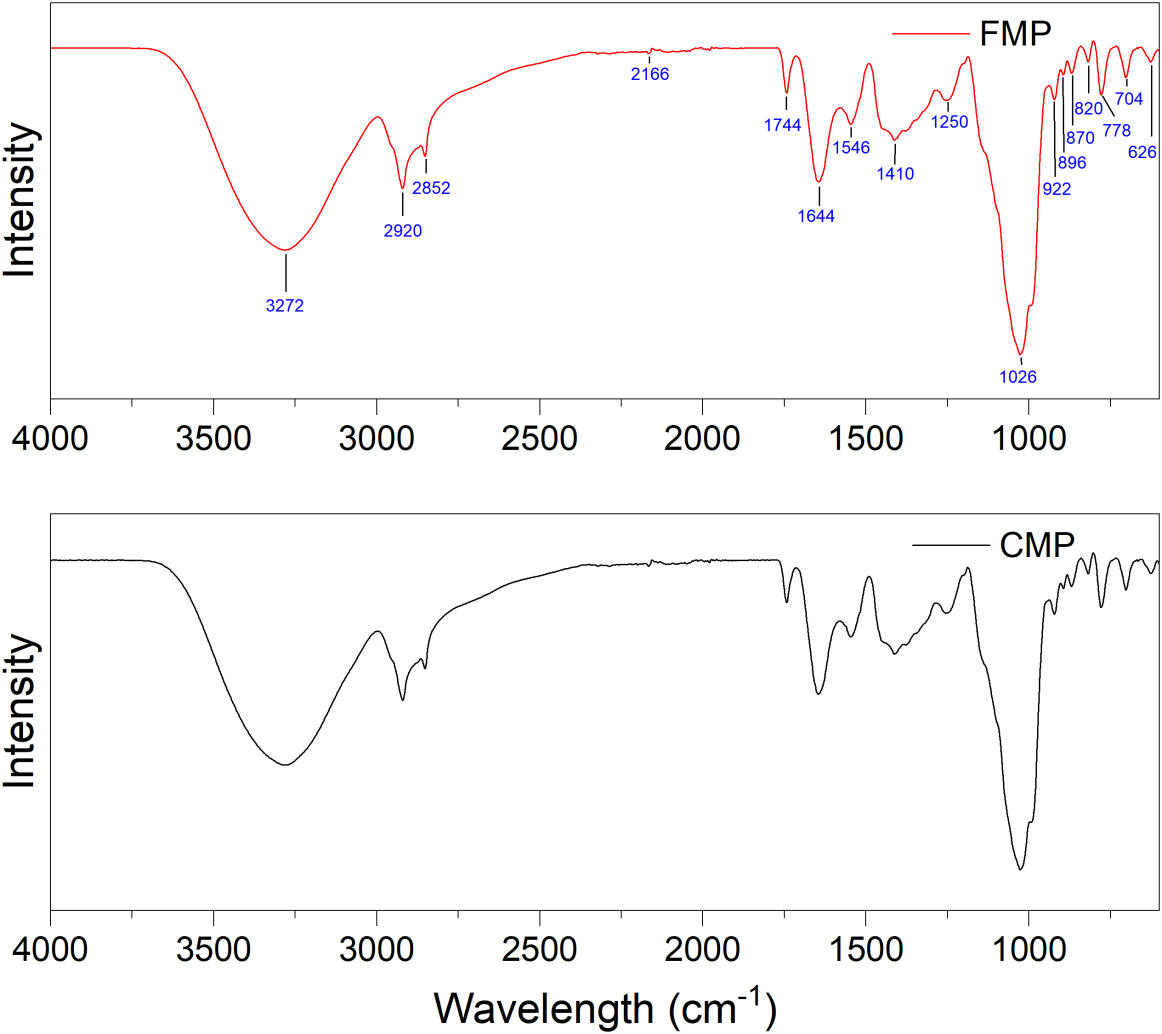
FTIR spectra of cashew apple juice‐fortified milk powder (FMP—red) and commercial milk powder (CMP—black).

FTIR spectroscopy provided insights into the chemical properties and interactions between spray‐dried CAJ‐fortified milk powder and commercial milk powder. The spectra unveiled striking similarities in the peaks of both milk powders (Figure 5), notably the presence of broad bands indicative of OH vibrations (3500–3000 cm^−1^), and the distinctive bands around 2900 cm^−1^ attributed to C–H bond stretching (Kalušević, Lević, Čalija, Pantić, et al., 2017). A prominent peak at 1026 cm^−1^ was evident in both types of milk powder, indicating the presence of –C–O linkages originating from polyphenols, ether, hydroxyl groups, and carbohydrates (when a drying agent, maltodextrin, is used). These results corroborated with the previously reported data (Ćujić‐Nikolić et al., 2019; De Souza et al., 2015). Additionally, subtle bands around this peak (1026 cm^−1^) could be attributed to organic acids from cashew apple juice. Bands in the 1600–1200 cm^−1^ range were likely associated with phenolic compounds in the CAJ‐fortified milk powder, while bands at 1644 and 1546 cm^−1^ were reported to amide I and amide II groups, respectively, indicating the presence of protein content in the milk (De Souza et al., 2015; Kalušević, Lević, Čalija, Pantić, et al., 2017; Kalušević, Lević, Čalija, Milić, et al., 2017). Based on the analysis, no new chemical interactions were found between the commercial milk powder and fortified CAJ milk powder after the spray drying.

## 4 CONCLUSION

This study systematically explores the spray drying of cashew apple juice–milk powder mixtures, using maltodextrin as a drying agent. To the best of our knowledge, no prior research has been conducted on spray‐dried cashew apple juice–milk powders. This work was intended to assess how the spray‐drying process affects the primary physical features of fortified cashew apple juice milk powders. The experiment, performed at optimal inlet and outlet temperatures, resulted in powders exhibiting desirable physical characteristics, such as adequate bulk density and good solubility. The results of this study indicate that maltodextrin is a suitable carrier material for facilitating the spray‐drying process of cashew apple juice‐blended milk powder. Varying the intake temperatures during spraying directly influenced the morphology of the powder, resulting in a higher number of particles with smooth surfaces and larger sizes, which was attributed to higher drying rates. The higher percentage of maltodextrin led to the formation of bigger particles due to the increased viscosity of the feed. The glass transition temperature, which was measured to be in the desirable range, further confirms the thermal stability of the product. Thus, it is strongly believed that the spray‐dried cashew apple juice‐fortified milk powder formulated in the current work has the potential to be used in dairy and other food avenues that produce flavored milkshakes, ice cream, yogurt, and cookies.

## AUTHOR CONTRIBUTIONS

**Vinoth Kannan Sithu Rameshbabu:** Conceptualization (lead); data curation (lead); investigation (lead); writing – original draft (lead); writing – review and editing (equal). **Vivek Rangarajan:** Supervision (equal); validation (equal); writing – review and editing (equal). **Sampatrao Dagu Manjare:** Funding acquisition (lead); resources (lead); supervision (equal); writing – review and editing (supporting).

## FUNDING INFORMATION

This research was funded by the Department of Science and Technology (DST) Goa (Grant number: 6‐198‐2016). We also acknowledge the Goa Energy Development Agency (GEDA) for the funding (sanction number: 1/426/GEDA/2023‐24/517).

## CONFLICT OF INTEREST STATEMENT

The authors declare that there is no conflict of interest associated with this article.

## Data Availability

The data presented in this study are available on request from the corresponding authors.
